# Analysis of Factors Affecting Patients' Compliance to Topical Antiglaucoma Medications in Egypt as a Developing Country Model

**DOI:** 10.1155/2015/234157

**Published:** 2015-06-17

**Authors:** Nahla B. Abu Hussein, Iman M. Eissa, Ahmed A. Abdel-Kader

**Affiliations:** Department of Ophthalmology, Faculty of Medicine, Cairo University, Cairo 11562, Egypt

## Abstract

*Purpose*. To study factors affecting patients' compliance to antiglaucoma medications in Egypt where there are unique factors as a developing country. *Patients and Methods*. A cross-sectional descriptive study on 440 Egyptian patients with open angle glaucoma (OAG) recruited for over two years. The patients were thoroughly interviewed about their age, education level, duration of glaucoma, difficulty in instilling the drops, medication regimens, a family history of glaucoma, knowledge of the disease, and the presence of medical insurance. *Results*. 236 (53.6%) were noncompliant compared to 204 (46.4%) who were compliant. Females had a tendency for higher compliance (*p*=0.061). Patient age above 50 years and low level of education and negative family history of glaucoma were factors significantly associated with poor compliance (*p* < 0.0001). Polytherapy and lack of medical insurance could be contributing factors. *Conclusion*. Egyptian patients have a high rate of noncompliance compared to the average in literature. Great effort is needed in educating patients about the importance of medications and the risk and the prognosis of this disease. Economic factors must also be taken into consideration in developing countries with large number of poor patients. We recommend simplifying drug regimens, incorporating electronic dose monitors, and creating reminders for follow-up visits of glaucoma patients.

## 1. Introduction

Glaucoma with both its entities, open angle glaucoma (OAG) and angle closure glaucoma (ACG), affected about 60.5 million people worldwide back in 2010. This number was estimated to increase to 79.6 million by 2020 [1].

There are many topical antiglaucoma medications, and most of them work through lowering intraocular pressure, the only treatable risk factor of glaucoma. Poor compliance with treatment is known to influence glaucoma progression [2, 3]. However, compliance to topical antiglaucoma medication has always been a major problem. This is greatly because treatment aims to stop or delay progression of the disease and there is absence of immediate visual restoration felt by the patient [4]. Diseases that are asymptomatic are more prone to poor patient compliance [5]. Patient sometimes assumes that the side effects of their drops imply the worsening of the ocular condition. Furthermore, ophthalmologists may mistake noncompliance for ineffectiveness of a given antiglaucoma medication and prescribe more medications or shift to surgery, aggravating the problem with additional costs and risks [6].

Many studies attributed compliance to factors like age, gender, level of education, and fear of blindness. Other factors include poor communication with the health care provider, cost of eye drops, forgetfulness, and difficulty in instilling the eye drops. Most of these studies agreed that compliance is a multifactorial complex behavior [7, 8].

Adherence to antiglaucoma medications is difficult to measure. This is because the patient usually overestimates his compliance level and usually sticks to the prescribed regimen two to three days prior to his next follow-up visit, so that even intraocular pressure cannot be considered a clue to patient adherence [9, 10]. In developing countries where socioeconomic standards are poor and the patients are not well-educated, if educated at all, it is even more challenging to measure patient compliance [11]. In Egypt, electronic dose monitors are not readily available, and pharmacy records are always missing or difficult to obtain because medical insurance does not cover most individuals, so the patient can buy his medication from whichever pharmacy he chooses and does not have to stick to certain pharmacies related to his insurance provider.

In this cross-sectional descriptive case study, we examined a sample of 440 Egyptian patients with chronic OAG aiming to investigate factors that may affect compliance and further understand the already existing ones expected to affect compliance especially in a developing country model like ours.

## 2. Patients and Methods

Four hundred and forty Egyptian patients were recruited equally from two major eye hospitals in Egypt for over two years from January 2012 to June 2014. One hospital serves mostly the poor from urban and rural areas (Kasr Al Aini University Hospital) and the other one (Al Oyoun International Eye Hospital) serves mostly urban patients with relatively higher socioeconomic standard. Inclusion criteria were patients above 18 years of age, with a specialist confirmed diagnosis of OAG, and those who have been on one or more topical antiglaucoma medications for at least one year. Exclusion criteria were patients who underwent glaucoma surgery as this was thought to be a motivator for compliance, non-Egyptian patients, or those whose intellectual level was too low to understand the interview questions, a desire of the patient to keep his data personal, and coexisting psychiatric disorders.

All participants were willing to be included in the study. An informed consent was obtained from all participants. The study protocol was approved by the Ethical Committee Board of Faculty of Medicine, Cairo University, Egypt. The study adhered to the guidelines of the Declaration of Helsinki.

One to one in depth interview was done by the same interviewer. The interview took around 20 minutes and was usually done on one of the patient's follow-up visits. The interviewer asked nonleading questions in an open discussion form not to stress the patient or lead him to certain answer models. The interviewer also tried to stress the value of accurate information rather than gain positive impression and shared the concept that failure to take medication as prescribed due to many personal, social, and economic factors is a common problem. The patient was asked about his age, duration of the disease, duration of treatment, dropper related difficulties, forgetting the eye drops, side effects related to the drops, the presence of any other systemic diseases, if the patient has ever attended school, highest education certificate or degree, knowledge about the disease, whether there is a family history of glaucoma, the last missed dose, frequency of missing a dose (was recorded as a percentage), and whether or not the patient has medical insurance.

Based on the patient's replies, a noncompliant patient was defined as one who omits over 10% of the weekly doses. This percentage was relatively strict (as opposed to the—albeit arbitrary—frequently used one in medicine, 20%) because patients tend to overestimate their self-reported adherence. Patients who cannot recall or show their medication forms and regimen of use were also recorded as being noncompliant. In order to decrease the subjectivity of our assessment, patients who gave responses that implied that they were compliant then received two unexpected phone calls at randomly chosen dates, which were at least 2 months apart. During the phone calls, we enquired about taking the last three doses of eye drops on time. Patients who answered “no” to any of the phone calls were no longer included in the “compliant” group. This occurred with eighteen patients (4.09%) who were no longer included in the compliant group. After a total period of almost two years of performing this interview and confirmation phone calls, the final data was collected and tabulated for statistical analysis.

Data were statistically described in terms of mean ± standard deviation (±SD) or frequencies (number of cases) and percentages when appropriate. Comparison of age between compliant and noncompliant groups was done using Student's *t*-test for independent samples. For comparing categorical data, Chi square (*χ*²) test was performed. Exact test was used instead when the expected frequency was less than 5. Univariate and multivariate logistic regression analysis models were used to test for the preferential effect of all the independent variable(s) on compliance. A *p* value less than 0.05 was considered statistically significant. All statistical calculations were done using the computer program SPSS (Statistical Package for Social Science; v15.0, SPSS Inc., Chicago, IL, USA).

## 3. Results

### 3.1. Gender and Age

Four hundred and forty patients were enrolled in our study. They included 143 (32.5%) females and 297 (67.5%) males (Table 1). The number of patients found to be noncompliant was 236 patients (53.6%), whereas 204 patients (46.4%) were found to be compliant to topical antiglaucoma medications.

In the female group, 78 patients (54.6% of females) were found to be compliant. In the male group, 126 patients (42.4% of males) were found to be compliant. Of all compliant patients, 38.2% were females and 61.8% were males, whereas 27.5% of noncompliant patients were females and 72.5% were males. Females showed significantly higher adherence to antiglaucoma medications (*p* = 0.017) in univariate analysis (Table 1). However, this significance dropped to (*p* = 0.061) upon multivariate analysis, with females only showing a tendency for higher compliance (Table 6).

The mean age of compliant group of patients was 49.77 years (±8.92 SD) and the mean age for noncompliant group was 54.24 years (±7.93 SD). Patients showed good compliance in age group below 50 years (66.17% of compliant patients), while 60.59% of noncompliant group aged above 50 years. This was statistically and highly significant (*p* < 0.001) in both univariate and multivariate analyses.

### 3.2. Level of Education and Knowledge about Glaucoma

Upon analyzing the effect of the level of education upon compliance (Table 2), we found a statistically and highly significant difference in compliance (*p* < 0.0001) between educated and noneducated patients, with the highest percentage of noncompliant patients (41.5%) falling in the noneducated (illiterate) group and the highest percentage of compliant patients (69.6%) falling in the group who finished high school and university graduates. This difference was highly significant (*p* < 0.001) in both univariate and multivariate analyses (Table 6).

Only 3.9% of our patients had proper knowledge about glaucoma disease describing it as a disease affecting the optic nerve and is commonly associated with, but not essentially, high IOP, with 53.8% of noncompliant patients having absolutely no understanding of the nature of glaucoma and 62.3% of compliant patients defining glaucoma merely as a rise in intraocular pressure. Other patients' knowledge about glaucoma was that it is a disease that causes “blue water” or “blue eye” which is the literal translation of glaucoma in Arabic (2.3%). Some defined glaucoma as optic atrophy (1.1%). Another statistically significant difference in compliance (*p* < 0.0001) was found between patients with proper knowledge about the disease and those with little or no understanding of the nature of the disease (Table 3). However, this difference was not statistically significant upon multivariate analysis with all other factors taken into consideration (*p* = 0.362) (Table 6).

### 3.3. Family History of Glaucoma

We had 208 (47.3%) of patients having a positive family history of glaucoma; 68.7% of them were compliant. While 232 (52.7%) of patients had negative family history of glaucoma, of them 26.3% were compliant. Another statistically and highly significant difference in compliance (*p* < 0.0001) was found between patients with a positive family history of glaucoma and patients giving no family history of glaucoma. This difference was still of high significance in multivariate analysis (*p* < 0.0001) (Table 6).

### 3.4. Dropper Related Difficulties and Physical Inability (Discompliance) and Side Effects of Medication

When studying “dropper related difficulties” and physical inability to instill drops, 87.7% of compliant patients had no reported dropper related difficulties, 11.3% reported difficulty in drop count, and 1% reported difficulty in squeezing the dropper. Of noncompliant patients, 72.9% had no dropper related difficulties, 26.3% reported difficulty in drop count, and 0.8% reported difficulty in squeezing the dropper. The difference between these groups was statistically significant (*p* < 0.001) in univariate analysis, but it was of no statistical significance (*p* = 0.483) with all variables tested against each other (Table 6).

Regarding eye drops related side effects such as itching, burning, redness, or systemic side effects, 64.3% of patients were suffering from side effects of medication. 60.6% of noncompliant patients reported side effects of medication while 68.6% of compliant group reported similar side effects. Medication side effects were of no role in compliance in both univariate and multivariate analyses (*p* = 0.187 and *p* = 0.801, resp.) (Table 6).

### 3.5. Laterality of Glaucoma, Number of Medications, and Frequency of Doses

Only 5.23% of our patients had unilateral glaucoma. 60.9% of them were compliant to glaucoma medication. However, laterality was a statistically insignificant factor of compliance (*p* = 0.108).  Table 4 shows that 218 (49.5%) of our patients were on monotherapy, with 51.8% of them showing compliance, 182 (41.4%) of our patients were on two drugs, with 41.8% of them being compliant, and 40 (7.3%) of our patients were on polytherapy, with 37.5% of them being compliant. Increasing the number of drugs seems to be associated with less compliance; however, this was of low significance statistically (*p* = 0.066).

Table 4 also shows that 47 (10.7%) of our patients were on once daily treatment, with 57.4% compliance, 362 of our patients were on twice daily treatment, with 45.6% compliance, and 31 (7.1%) of our patients were on three or more times daily treatment, with 38.7% compliance. However, the frequency of treatment was not statistically significant (*p* = 0.355).

### 3.6. Systemic Comorbidity

51% of compliant patients had no coexisting morbidity as opposed to 24.6% of noncompliant patients. High percentages of noncompliant patients had hypertension (31.4%), diabetes with hypertension (16.5%), and ischemic heart disease (13.1%). Systemic comorbidity had a statistically significant association with compliance (*p* < 0.0001). However, systemic comorbidity played minor role in noncompliance (*p* = 0.147) when multivariate regression analysis was done (Table 6).

### 3.7. Medical Insurance

231 (52.5%) of our patients were covered by medical insurance, with 51.9% of them falling in the compliant group, while 209 (47.5%) of our patients were not medically insured, with 40.19% of them being compliant. By medical insurance we mean that the cost of eye drops was totally covered. Most insured subjects worked for private and/or multinational companies. Medical insurance was found to have a statistically significant association with compliance (*p* = 0.017) in univariate analysis (Table 5) but not in multivariate analysis (*p* = 0.134) (Table 6).

### 3.8. Duration of the Disease and Last Follow-Up Visit

The mean duration of glaucoma in compliant group was 3.34 years (±2.09 SD) and in the noncompliant group of patients it was 3.17 years (±1.9 SD). Duration of disease was found to be of a statistically insignificant association with compliance (*p* = 0.358). However, the last visit of followup was 7.24 months (±5.13 SD) in the compliant group compared to 12.02 months (±10.17 SD) in the noncompliant group. Thus, noncompliance to medication was found to be associated with poor compliance to follow-up visits (*p* < 0.0001).

## 4. Discussion

Glaucoma is the second leading cause of blindness worldwide as defined by the World Health Organization (WHO) [12]. Few regional studies have defined glaucoma as the leading cause of blindness at a prevalence rate of 2–9% in Egypt, 1.73% in Qatar, and 4.75% in Oman [13–15]. Glaucoma had also been found to contribute to legal blindness at a rate of 8% in a study done at Cairo University in 2014, 12.1% at Mansoura University in 2002, 19.7% at Alexandria University in 1987, and 7.6% at Al-Azhar University (Cairo) in 1989 [16].

Noncompliance with medical therapy has long been recognized as an important limiting factor in the medical management of any chronic disease [17]. Patients with glaucoma who have lower rates of compliance are presumed to be at greater risk of developing visual loss [18]. Our cross-sectional descriptive study was done to evaluate factors affecting compliance to glaucoma medications among a sample of Egyptian population as a part of the African, Arab, Middle-East, and developing countries. To the best of our knowledge, this is the largest reported regional case based study involving 440 patients. In our study, rather than studying “situational and patient factors as forgetfulness, travel, busy schedule, social responsibilities, and so on,” we evaluated factors with a possible impact on compliance or factors that we believed can be corrected or improved to increase adherence to glaucoma medication.

In our study, we had a high percentage of noncompliance to glaucoma medication (53.6%) that frames the magnitude of the problem. In literature, a meta-analysis found that noncompliance ranges from 5 to 80% for glaucoma patients, although definitions and methods of evaluating compliance were not standardized [19]. Patel and Spaeth reported that 59% of glaucoma patients were not strictly compliant [20]. A noncompliance rate of 75.2% was reported among Oman glaucoma population in 2005 [21]. In our study, higher noncompliance was found in elderly patients above 50 years old. Older patients may have a lower compliance probably due to the lack of family support, reduced vision, problems with manual dexterity, coordination, comprehension, or memory; however, this was not evaluated in this study. Gender, dropper related difficulties, duration of the disease, medication side effects, and systemic comorbidity did not have a significant association with compliance based on our multivariate analysis. We found major association between high education level, proper knowledge of the disease, and better compliance. 74.9% of patients with education below high school level were noncompliant and 73.6% of patients with education above high school were compliant. We had 41.4% of our patients literally not understanding what is glaucoma, with 69.8% of them being noncompliant. This data validates other studies [22–24]. Norell in 1979, Rendell in 2000, and Okeke et al. in 2009 found that improving knowledge about glaucoma through education significantly improved compliance [25–27]. However, in our study, multivariate analysis and lack of formal education had a much more statistically significant association with compliance than knowledge about the disease (Table 6).

Another factor that may affect compliance in our study was financial coverage of cost of therapy through medical insurance. Univariate analysis showed statistically significant higher compliance rate among insurance covered patients. Eldaly et al. showed that lack of IOP control is mostly related to the economic burden of glaucoma medications in Egypt [11].

Our study showed a statistically and highly significant compliance in patients with a positive family history of glaucoma, which may again point to a better compliance with knowledge about the disease that is acquired from family. We also found that poor compliance was associated with poor attendance of follow-up visits rather than the duration of the disease (no show = no drops) [28]. Gurwitz et al. reported that noncompliance is most strongly related to < 2 visits, with an ophthalmologist within a 1-year study [29].

Our study pointed the important effect of number of medications as well as the complexity of regimen on compliance rate among glaucoma patients, which agrees with many studies [30–33]. However, our data only showed a tendency to being statistically significant.

Assessing compliance in ocular treatment is difficult compared to other medical therapies. In other systemic medication, compliance could be assessed by blood level of the medication in addition to the response to treatment [34]. Intraocular pressure (IOP) assessment as an indicator of compliance is associated with “white coat syndrome” with a percentage of patients adhering to their medication regimen in the days preceding office visit and declining thereafter [35]. Okeke et al. showed that patients are more compliant just before and just after office visit, with 55% of patients taking 75% of their required drops [36].

Limitations of our study include being a questionnaire-based study and there is a possibility of patients' underreporting missed doses due to recall bias and/or a desire to please the physician with inaccurate estimation of compliance rate. However, we tried to do an audit of the questionnaire through the recheck phone calls to improve accuracy and double check the patient's claim of compliance. We did not use the criterion of never missing a dose for compliance, as this would reveal most of our subjects being noncompliant. Instead, we used the criterion of missing more than 10% of the collective weekly regimen. However, we did not evaluate the effect of that on the progression of the disease.

Electronic medication monitoring (Medication Events Monitoring Systems “MEMS”) may have been proved to be an accurate estimate of patients' compliance as demonstrated by Kass et al. showing that interviewed patients state 90% compliance, physicians estimated 79% compliance, and electronic eye drop monitoring shows 75% compliance. In addition, electronic monitoring has the advantage of providing information on date and time of each dose [10]. However, electronic monitoring has the limitations of being technically difficult and patients' awareness of being monitored could change their behavior of simply being observed: “the Hawthorne effect.” Also electronic devices record drop dispension but do not confirm placement in the eye.

Using pharmacy claims of refill data as an indicator of compliance had been tried in Glaucoma Adherence and Persistency Study, GAPS [37]. Gurwitz et al. found 24.7% noncompliance using prescription collection data [29]. However, pharmacy claims are not available in our country systems. In addition, pharmacy claims evaluating Medication Possession Ratio (MPR) gauge patients who are getting eye drops in hand but cannot ensure if eye drops are being used properly. It also underestimates patients with unilateral disease and those receiving free samples.

## 5. Conclusion and Recommendations

Our study revealed a high noncompliance rate among Egyptian glaucoma patients compared to the average of other studies in developed world. It is mostly a multifactorial problem. Higher formal education and a positive family history of glaucoma were the main associations with good compliance. Poor understanding of the disease may lead to poor compliance. This is a global problem that needs cooperation of physicians, media, and social care providers. Extra effort needs to be done by health care providers to educate our patients about the nature of glaucoma, glaucoma susceptibility, importance of treatment, follow-up visits, and effect of treatment on prognosis. Longer time has to be spent with our patients teaching them how to instill their drops. Also, simplifying treatment regimen and tailoring it to their daily routine lifestyle are a must. Reminders of follow-up visits with proper tracking of our patients must be added to our health care system. We also need to incorporate better compliance evaluation methods as electronic drug monitoring for evaluating behavioral changes in our patients' utilization of medical treatment.

## Figures and Tables

**Table 1:** Age & gender characteristics and compliance to antiglaucoma medication.

Age & sex	Compliance		Total
	No	Yes	
Age (mean ± SD)	54.24 ± 7.93	49.77 ± 8.92	
Female	65 (27.5%)	78 (38.2%)	143 (32.5%)
Male	171 (72.5%)	126 (61.8%)	297 (67.5%)

Total	236 (53.6%)	204 (46.4%)	440

**Table 2:** Effect of formal education level on compliance to antiglaucoma medication.

Education	Compliance		Total
	No	Yes	
None	98 (41.5%)	17 (8.3%)	115 (26.1%)
Elementary	84 (35.6%)	39 (19.1%)	123 (28%)
Middle	3 (1.3%)	6 (2.9%)	9 (2%)
High school	40 (16.9%)	94 (46.1%)	134 (30.5%)
University	11 (4.7%)	48 (23.5%)	59 (13.4%)

Total	236 (53.6%)	204 (46.4%)	440

**Table 3:** Effect of basic knowledge of glaucoma disease on compliance to antiglaucoma medication.

Knowledge about disease	Compliance		Total
	No	Yes	
High IOP/optic atrophy	1 (0.4%)	16 (7.8%)	17 (3.9%)
High IOP	97 (41.1%)	127 (62.3%)	224 (50.9%)
High IOP/blue eye	2 (0.8%)	0	2 (0.5%)
Blue eye	6 (2.5%)	4 (2%)	10 (2.3%)
None	127 (53.8%)	55 (27%)	182 (41.4%)

Total	236 (53.6%)	204 (46.4%)	440

**Table 4:** Effect of number of medications and frequency of drops on compliance to antiglaucoma medication.

	Compliance		Total
	No	Yes	
Number of medications			
1	105 (44.5%)	113 (55.4%)	218 (49.5%)
2	106 (44.9%)	76 (37.3%)	182 (41.4%)
≥3	25 (10.6%)	15 (7.3%)	40 (9%)
Frequency			
1	20 (8.5%)	27 (13.2%)	47 (10.7%)
2	197 (83.5%)	165 (80.9%)	362 (82.3%)
≥3	19 (8%)	12 (5.9%)	31 (7%)

Total	236 (53.6%)	204 (46.4%)	440

**Table 5:** Effect of medical insurance on compliance to antiglaucoma medications.

Medical insurance	Compliance		Total
	No	Yes	
No	125 (53%)	84 (41.2%)	209 (47.5%)
Yes	111 (47%)	120 (58.8%)	231 (52.5%)

Total	236 (53.6%)	204 (46.4%)	440

**Table 6:** Multivariate logistic regression analysis of factors evaluated for association with noncompliance to antiglaucoma medication.

Factor	*B*	S.E.	Wald	df	*p* value
Sex	0.485	0.259	3.501	1	0.061
Age	−0.038	0.007	26.750	1	<0.001
Education	0.516	0.101	25.932	1	<0.0001
Knowledge	0.293	0.321	0.832	1	0.362
Family history	0.993	0.259	14.659	1	<0.0001
Discompliance	0.251	0.359	0.492	1	0.483
Medication side effects	0.074	0.294	0.063	1	0.801
Systemic diseases	−0.371	0.255	2.105	1	0.147
Insurance	0.421	0.281	2.249	1	0.134

## References

[1] Quigley H., Broman A. T. (2006). The number of people with glaucoma worldwide in 2010 and 2020. *British Journal of Ophthalmology*.

[2] Rossi G. C. M., Pasinetti G. M., Scudeller L., Radaelli R., Bianchi P. E. (2011). Do adherence rates and glaucomatous visual field progression correlate?. *European Journal of Ophthalmology*.

[3] Sleath B., Blalock S., Covert D. (2011). The relationship between glaucoma medication adherence, eye drop technique, and visual field defect severity. *Ophthalmology*.

[4] Kass M. A., Heuer D. K., Higginbotham E. J. (2002). The Ocular Hypertension Treatment Study: a randomized trial determines that topical ocular hypotensive medication delays or prevents the onset of primary open-angle glaucoma. *Archives of Ophthalmology*.

[5] Dimatteo M. R., Giordani P. J., Lepper H. S., Croghan T. W. (2002). Patient adherence and medical treatment outcomes: a meta-analysis. *Medical Care*.

[6] Van Buskirk E. M. (1986). The compliance factor. *American Journal of Ophthalmology*.

[7] Lacey J., Cate H., Broadway D. C. (2009). Barriers to adherence with glaucoma medications: a qualitative research study. *Eye*.

[8] Friedman D. S., Hahn S. R., Gelb L. (2008). Doctor-patient communication, health-related beliefs, and adherence in glaucoma. Results from the Glaucoma Adherence and Persistency study. *Ophthalmology*.

[9] Kass M. A., Gordon M., Meltzer D. W. (1986). Can ophthalmologists correctly identify patients defaulting from pilocarpine therapy?. *The American Journal of Ophthalmology*.

[10] Kass M. A., Meltzer D. W., Gordon M., Cooper D., Goldberg J. (1986). Compliance with topical pilocarpine treatment. *American Journal of Ophthalmology*.

[11] Eldaly M., Hunter M., Khafagy M. (2007). The socioeconomic impact among Egyptian glaucoma patients. *British Journal of Ophthalmology*.

[12] Bourne R. R. A. (2006). Worldwide glaucoma through the looking glass. *British Journal of Ophthalmology*.

[13] Said M. E., Goldstein H., Korra A., el-Kashlan K. (1971). Visual acuity as related to causes of blindness, age and sex in urban and rural Egyptians. *The American Journal of Public Health*.

[14] Al-Mansouri F., Kanaan A., Gamra H. (2011). Prevalence and determinants of glaucoma in citizens of Qatar aged 40 years or older: a community-based survey. *Middle East African Journal of Ophthalmology*.

[15] Khandekar R., Jaffer M. A., Al Raisi A. (2008). Oman eye study 2005: prevalence and determinants of glaucoma. *Eastern Mediterranean Health Journal*.

[16] Eldaly M. A., Salama M. M., Abu Eleinen K. G., Ghalwash D., Youssef M., El-Shiaty A. F. (2014). Blindness and visual impairment among Egyptian glaucoma patients. *Journal of Ophthalmology*.

[17] Rudd P. (1979). In search of the gold standard for compliance measurement. *Archives of Internal Medicine*.

[18] Kass M. A. (1985). Compliance and prognosis in glaucoma. *Archives of Ophthalmology*.

[19] Olthoff C. M. G., Schouten J. S. A. G., van de Borne B. W., Webers C. A. B. (2005). Noncompliance with ocular hypotensive treatment in patients with glaucoma or ocular hypertension: An evidence-based review. *Ophthalmology*.

[20] Patel S. C., Spaeth G. L. (1995). Compliance in patients prescribed eyedrops for glaucoma. *Ophthalmic Surgery*.

[21] Khandekar R., Shama M. E.-S., Mohammed A. J. (2005). Noncompliance with medical treatment among glaucoma patients in Oman—a cross-sectional descriptive study. *Ophthalmic Epidemiology*.

[22] Stryker J. E., Beck A. D., Primo S. A. (2010). An exploratory study of factors influencing glaucoma treatment adherence. *Journal of Glaucoma*.

[23] Lunnela J., Kääriäinen M., Kyngäs H. (2010). The views of compliant glaucoma patients on counselling and social support. *Scandinavian Journal of Caring Sciences*.

[24] Deokule S., Sadiq S., Shah S. (2004). Chronic open angle glaucoma: patient awareness of the nature of the disease, topical medication, compliance and the prevalence of systemic symptoms. *Ophthalmic and Physiological Optics*.

[25] Norell S. E. (1979). Improving medication compliance: a randomised clinical trial. *British Medical Journal*.

[26] Rendell J. (2000). Effect of health education on patients' beliefs about glaucoma and compliance. *Insight*.

[27] Okeke C. O., Quigley H. A., Jampel H. D. (2009). Interventions improve poor adherence with once daily glaucoma medications in electronically monitored patients. *Ophthalmology*.

[28] Kosoko O., Quigley H. A., Vitale S., Enger C., Kerrigan L., Tielsch J. M. (1998). Risk factors for noncompliance with glaucoma follow-up visits in a residents' eye clinic. *Ophthalmology*.

[29] Gurwitz J. H., Yeomans S. M., Glynn R. J., Lewis B. E., Levin R., Avorn J. (1998). Patient noncompliance in the managed care setting. The case of medical therapy for glaucoma. *Medical Care*.

[30] Robin A. L., Novack G. D., Covert D. W., Crockett R. S., Marcic T. S. (2007). Adherence in glaucoma: objective measurements of once-daily and adjunctive medication use. *American Journal of Ophthalmology*.

[31] Robin A. L., Covert D. (2005). Does adjunctive glaucoma therapy affect adherence to the initial primary therapy?. *Ophthalmology*.

[32] Stewart W. C., Konstas A. G. P., Pfeiffer N. (2004). Patient and ophthalmologist attitudes concerning compliance and dosing in glaucoma treatment. *Journal of Ocular Pharmacology and Therapeutics*.

[33] Konstas A. G. P., Maskaleris G., Gratsonidis S., Sardelli C. (2000). Compliance and viewpoint of glaucoma patients in Greece. *Eye*.

[34] Cramer J. A. (2002). Effect of partial compliance on cardiovascular medication effectiveness. *Heart*.

[35] Feinstein A. R. (1990). On white-coat effects and the electronic monitoring of compliance. *Archives of Internal Medicine*.

[36] Okeke C. O., Quigley H. A., Jampel H. D. (2009). Adherence with topical glaucoma medication monitored electronically the Travatan Dosing Aid study. *Ophthalmology*.

[37] Friedman D. S., Quigley H. A., Gelb L. (2007). Using pharmacy claims data to study adherence to glaucoma medications: methodology and findings of the Glaucoma Adherence and Persistency Study (GAPS). *Investigative Ophthalmology and Visual Science*.

